# Assessing Mixed Quantum-Classical Molecular Dynamics Methods for Nonadiabatic Dynamics of Molecules on Metal Surfaces

**DOI:** 10.1021/acs.jpcc.3c03591

**Published:** 2023-07-28

**Authors:** James Gardner, Scott Habershon, Reinhard J. Maurer

**Affiliations:** † Department of Chemistry, University of Warwick, Gibbet Hill Road, Coventry CV4 7AL, United Kingdom; ‡ Department of Physics, University of Warwick, Gibbet Hill Road, Coventry CV4 7AL, United Kingdom

## Abstract

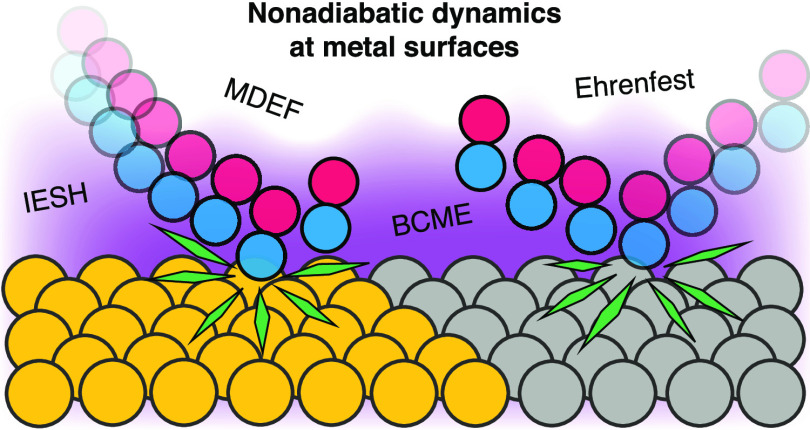

Mixed quantum-classical (MQC) methods for simulating the dynamics of molecules at metal surfaces have the potential to accurately and efficiently provide mechanistic insight into reactive processes. Here, we introduce simple two-dimensional models for the scattering of diatomic molecules at metal surfaces based on recently published electronic structure data. We apply several MQC methods to investigate their ability to capture how nonadiabatic effects influence molecule–metal energy transfer during the scattering process. Specifically, we compare molecular dynamics with electronic friction, Ehrenfest dynamics, independent electron surface hopping, and the broadened classical master equation approach. In the case of independent electron surface hopping, we implement a simple decoherence correction approach and assess its impact on vibrationally inelastic scattering. Our results show that simple, low-dimensional models can be used to qualitatively capture experimentally observed vibrational energy transfer and provide insight into the relative performance of different MQC schemes. We observe that all approaches predict similar kinetic energy dependence but return different vibrational energy distributions. Finally, by varying the molecule–metal coupling, we can assess the coupling regime in which some MQC methods become unsuitable.

## 1 Introduction

When atoms and molecules adsorb and react at metal surfaces, they lose kinetic energy by directly exciting electron–hole pair excitations in the metal. Several seminal experimental works have shown the strong impact of nonadiabatic coupling and hot electron effects on experimentally measurable signatures of surface chemistry.^1−3^ By establishing a deep understanding of nonadiabatic dynamics at metal surfaces, new applications and technologies that utilize nonadiabatic energy transfer can be developed for catalysis and energy conversion, such as light- and hot-carrier-driven chemistry on plasmonic metal nanostructures.^4,5^ Achieving insight into an atomistic level requires computational simulation methods that can accurately describe nonadiabatic effects during dynamics while scaling efficiently for realistic systems. The simulation of the experimentally measurable reaction and scattering probabilities requires statistically significant averages over many tens of thousands of simulation events. Classical molecular dynamics (MD) simulations have proven effective at treating systems where the adiabatic approximation is valid, but going beyond MD to include nonadiabatic effects is a challenging task.^6,7^ Many approximate methods that treat electrons quantum mechanically and atoms classically, so-called mixed quantum-classical (MQC) methods, have been proposed for the description of coupled electron–nuclear dynamics at metal surfaces, including molecular dynamics with electronic friction (MDEF),^8−14^ independent electron surface hopping (IESH),^15−20^ and classical master equation (CME) surface hopping.^21−25^

An ongoing challenge for simulating nonadiabatic dynamics at surfaces lies in the reliability of different simulation techniques.^26^ Often, it is difficult to know if the simulations are correctly describing reality as accurate reference results are rare. Progress has been made in this area from two directions, namely, verifying methods against quantum dynamics for simple analytical model Hamiltonians^10,18,26,27^ and comparing the outcomes of high-dimensional simulations, often based on first-principles electronic structure theory, to experimental observations.^3,13,28−32^ Both approaches have limitations. The former approach may unduly simplify the electronic structure and the influence of many coupled, anharmonic degrees of freedom. The latter makes it difficult to disentangle errors that arise from the electronic structure description and errors that are intrinsic to the approximations of the applied MQC method. For example, Shenvi et al.^15^ have applied the independent electron surface hopping method to study nonadiabatic vibrational energy loss during nitrous oxide (NO) scattering on Au(111) and they found that the method was able to describe dynamical steering effects connected to vibrational energy loss. Later, it was shown that the employed potential energy landscape based on density functional theory (DFT) misrepresented energy barriers, which led to an incorrect description of the translational energy dependence of vibrational inelasticity during scattering.^32^ While previous works questioned the ability of molecular dynamics with electronic friction to describe vibrational energy loss for this system, a new and improved potential energy landscape enabled an accurate description with the MDEF method, at least for the case of low vibrational incidence energy.^13^ Hyperthermal scattering of NO from Au(111) and Ag(111) remains one of the most studied experimental reference systems to understand nonadiabatic effects in surface chemistry.^33,34^ As a quantum reference, the hierarchical quantum master equations (HQMEs) promise a numerically exact treatment of coupled electron-vibrational systems;^35−41^ however, the method is currently limited to only a few degrees of freedom, which precludes an extension to large atomistic systems. Without a scalable reference method, it is difficult to bridge the gap between simple models and complex systems, casting continued uncertainty on the validity of approximate MQC methods.

Another limitation in the development of MQC methods is the lack of model systems that can be related to realistic counterparts. The ubiquitous models introduced by Tully^42^ have been used countless times in recent decades to benchmark and compare methods for nonadiabatic dynamics^43−54^ and have been recently shown to closely relate to real molecular systems.^54^ However, similar models for dynamics at metal surfaces are less widespread.^55^ A unified collection of models that are capable of relating to experimentally measurable phenomena would be beneficial for the further development of MQC methods.

In this work, we apply MDEF, IESH, Ehrenfest dynamics, broadened classical master equation (BCME), and adiabatic MD to two-dimensional model Hamiltonians that describe the scattering of diatomic molecules on metal surfaces. The two models introduced are designed to have a simple analytic form for easy implementation and usage while closely matching recently published ground- and excited-state ab initio potential energy surfaces (PESs) to ensure that the models are physically relevant.^56^ Using these models, we explore the effect of decoherence on molecular scattering, as modeled by IESH, and find that decoherence can have subtle effects on vibrational energy transfer during molecular scattering. Furthermore, we compare the full set of MQC methods and determine that all methods capture similar trends in kinetic energy dependence for models that feature realistic model parameters but deviate in the widths of the vibrational distributions. By exploring models with stronger and weaker molecule–metal coupling than what is observed experimentally, we identify the limitations of the respective MQC methods.

Much previous work has focused on the electron-transfer problem in a harmonic double-well, within the wide-band limit, where Marcus theory can be used as a benchmark.^18,19,22^ However, rarely has the case been explored where the molecule–metal coupling depends on the molecular coordinates, where the wide-band limit is less well-defined.^25^ A key novelty of our new models is that they are inspired by ab initio data in order to capture vibrational de-excitation during nonadiabatic scattering. This allows us to study in greater detail the case in which the coupling strength depends on the molecule–metal distance.

The outline of this article is as follows. In Section 2, we introduce the Newns–Anderson Hamiltonian (NAH) and MQC methods used for the simulations. Section 3.1 presents the parameterization of two models based on the well-studied NO on Au(111) and NO on Ag(111) systems and Section 3.2 reports the computational details of the simulations. Section 3.3 explores the effect of decoherence in IESH for scattering problems, and Section 3.4 compares the performance of the MQC methods. In Section 3.5, the coupling strength is modified to investigate how the performance of each method changes. Section 4 closes the article with our conclusions.

## 2 Theory

### 2.1 Newns–Anderson Hamiltonian

The standard model for nonadiabatic dynamics at metal surfaces is the NAH, written as
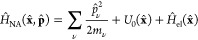
1where **x̂** is the vector of nuclear coordinate operators and **p̂** is their conjugate momenta with particle masses **m**. The index ν is used to label each nuclear degree of freedom in the system. The electronic-state-independent potential energy function *U*_0_ and the electronic Hamiltonian  determine the potential energy of the system. The electronic Hamiltonian for a discretized metallic continuum of states is
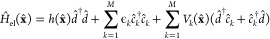
2where *d̂*^†^(*d̂*) are the creation (annihilation) operators for an electron in the molecular state and *ĉ*_k_^†^(*ĉ_k_*) are the creation (annihilation) operators for an electron in the metal state *k*. When the molecular state is occupied, *h*(*x*) = *U*_1_(**x̂**) − *U*_0_(**x̂**) is added as a further contribution to the system potential energy. To obtain the coupling terms *V_k_* that allow population transfer between the metal and molecule, it is necessary to discretize the hybridization function
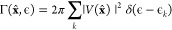
3In this work, the problem is simplified using the wide-band approximation such that the hybridization function becomes independent of energy Γ(**x̂**, ϵ) = Γ(**x̂**). The coupling terms then become , where the weights *w_k_* can be obtained using different discretization methods.^20^

### 2.2 Mixed Quantum-Classical Dynamics Methods

MQC dynamics methods allow for the simulation of coupled nuclear-electronic dynamics at metal surfaces. The treatment of the nuclei as classical particles ensures scalability and computational efficiency, improving the ability of the MQC methods to treat complex systems that are not tractable using quantum dynamics methods. However, using classical nuclei precludes the treatment of nuclear quantum effects. In this paper, we only consider classical nuclear motion. The following sections briefly introduce the methods that are used for the simulations in Section 3.

#### 2.2.1 Molecular Dynamics with Electronic Friction

One of the most popular methods for simulating dynamics at surfaces is MDEF, which captures electron–nuclear coupling via a system-bath description using a Langevin equation.^8,9,12,14,57^ The key ingredient of MDEF is the friction tensor, which governs the transfer of energy between the nuclei and electrons. Although obtaining the friction tensor can be challenging using ab initio calculations,^9,57,58^ for the NAH in the wide-band limit, the exact friction tensor is given by^59,60^
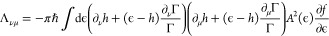
4where
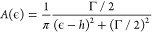
5with ∂_ν_ = ∂/∂*x*_ν_ and ∂*f*/∂ϵ being the gradient of the Fermi function.

The MDEF equations of motion for the NAH can be written as
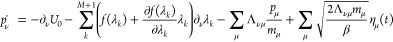
6The first two terms arise due to the adiabatic force where {*λ_k_*} are the *M* + 1 eigenvalues of the electronic Hamiltonian (*M* metallic states and 1 molecule state) and *f*(*λ_k_*) is the Fermi function that ensures a thermal contribution from each state. The third term is the retarding force that transfers energy from the nuclei to the electrons. The fourth and final term is the random force component that ensures the equations of motion correctly recover thermal equilibrium, where η*_μ_*(*t*) is a Gaussian-distributed random number with zero mean and unit variance.

#### 2.2.2 Independent Electron Surface Hopping

IESH^16−18,20^ models coupled nuclear-electronic dynamics near metal surfaces by coupling a finite set of discretized electronic states in the metal with the molecular state. The electrons in the system are propagated independently in time, and the coupling between electrons and nuclear degrees of freedom is described via stochastic hops that represent electronic transitions. Previously, IESH has been used to investigate the scattering of NO on Au(111),^15,30^ calculate electron-transfer rates,^18^ and describe desorption and scattering in a one-dimensional model.^20^ The nuclear dynamics in IESH are governed by the Hamiltonian
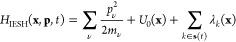
7where **s**(*t*) is the vector that contains the indices of states occupied by electrons, such that the summation includes only occupied states. From this Hamiltonian, it is clear that the nuclei evolve on a potential determined by the electronic occupations at each point in time. The electronic occupations change during the dynamics by allowing a single electron to hop each time step with probabilities obtained from the usual criteria of Tully’s fewest-switches surface hopping.^42^ In order to calculate the hopping probabilities, it is necessary to propagate the electronic wave functions for each electron alongside the nuclear dynamics by solving the time-dependent electronic Schrödinger equation
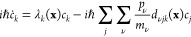
8where {*c_k_*} are the complex expansion coefficients for each electron and *d*_ν_*_jk_* is the nonadiabatic coupling along coordinate ν between adiabatic states *j* and *k*. The electronic coefficients are initialized such that they are consistent with the discrete occupations.

#### 2.2.3 Ehrenfest Dynamics

The Ehrenfest dynamics method allows the nuclei to evolve on the PES obtained from the expectation value of the electronic Hamiltonian.^6,61−63^ The Hamiltonian that describes Ehrenfest dynamics for the nuclei based on a Newns–Anderson Hamiltonian, *Ĥ*_el_, is
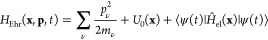
9where ψ(*t*) is the electronic wave function at time *t*. As with independent electron surface hopping, the electronic wave function is coherently propagated alongside the nuclear dynamics using eq 8. However, unlike both MDEF and IESH, the Ehrenfest method is entirely deterministic, such that each trajectory is uniquely determined by its initial conditions.

#### 2.2.4 Broadened Classical Master Equation

Another alternative is to model the presence of the electronic bath implicitly by representing the dynamics with a classical master equation that describes the time evolution of the nuclear probability density of the system.^21,22^ The CME method involves classical dynamics on a single diabatic state with transitions between states that ensure the correct thermal equilibrium is reached when Γ is small. The original limitation to the regime of small Γ was due to the neglect of broadening effects induced by the molecule–metal coupling. To go beyond the regime of small Γ, broadening effects were previously incorporated by extrapolating the CME forces to the adiabatic regime. The BCME recovers the original CME when Γ is small but yields adiabatic dynamics on a broadened potential of mean force when Γ is large.^24,25^ In addition to the modified force, the original proposal for BCME included momentum jumps in the algorithm.^24^ However, later, an alternative form was introduced with slightly modified forces that no longer required any momentum jumps.^25^

The updated form of the broadened master equation is^26^
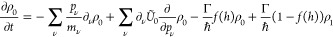
10
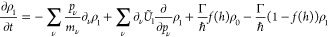
11where
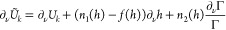
12and
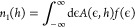
13
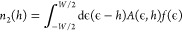
14The two broadening functions *n*_1_ and *n*_2_ involve a convolution of the Fermi function *f*(ϵ) with a Lorentzian function
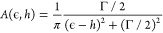
15

The final term in eq 12 involving ∂Γ/∂*x_i_* was proposed as an additional contribution to the force that includes non-Condon effects in the BCME dynamics.^25^ However, the integral in eq 14 diverges logarithmically in the wide-band limit where *W* → ∞.^23,25^ Therefore, whenever ∂Γ/∂*x_i_* is nonzero, the potential of mean force will depend on the width of the band.

## 3 Results and Discussion

### 3.1 Models

The simulations in this paper are focused on two analytical models that describe the interaction of a NO molecule with two different metal surfaces: Au and Ag. We consider only two degrees of freedom: the center of mass distance between the molecule and surface *z* and the intramolecular distance *r*. In our models, we assume that the molecular axis is always aligned perpendicular to the surface and that the N atom always faces down. The form of the two diabatic potential energy surfaces is chosen to be
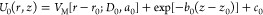
16
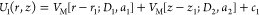
17where *V*_M_ is the Morse potential defined as
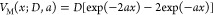
18The coupling is chosen to be dependent on only the molecule-surface distance, given by
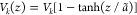
19The decision has been made to restrict the models to a simple analytic form to ensure that the models can be easily understood and implemented. With the functional form of the models established, it is necessary to choose values for each of the parameters in eqs 16, 17, and 19. The neutral *U*_0_ bond stretching Morse parameters are taken from Laporta et al.^64^ To ensure that the models best represent the molecule–metal interaction, the remaining parameters for *U*_0_ and *U*_1_ have been fitted to the density functional theory data presented by Meng and Jiang.^56^ Meng and Jiang^56^ employed constrained density functional theory (CDFT) to model the scenario where the molecule does not exchange charge with the surface (*U*_0_) and where the molecule accepts a full electron from the surface (*U*_1_). They presented the adiabatic and diabatic potential energy surfaces for several one-dimensional curves (Figures 7 and 9 in ref (56)) along *z* and *r* for each of the two metal surfaces, Au(111) and Ag(111), with the molecule laterally placed in an hcp site with the N atom facing down. Using the reference data, eqs 16 and 17 have been fitted using the gradient-free Nelder–Mead method^65^ as implemented in the *Optim.jl* package.^66,67^

**Figure 1:**
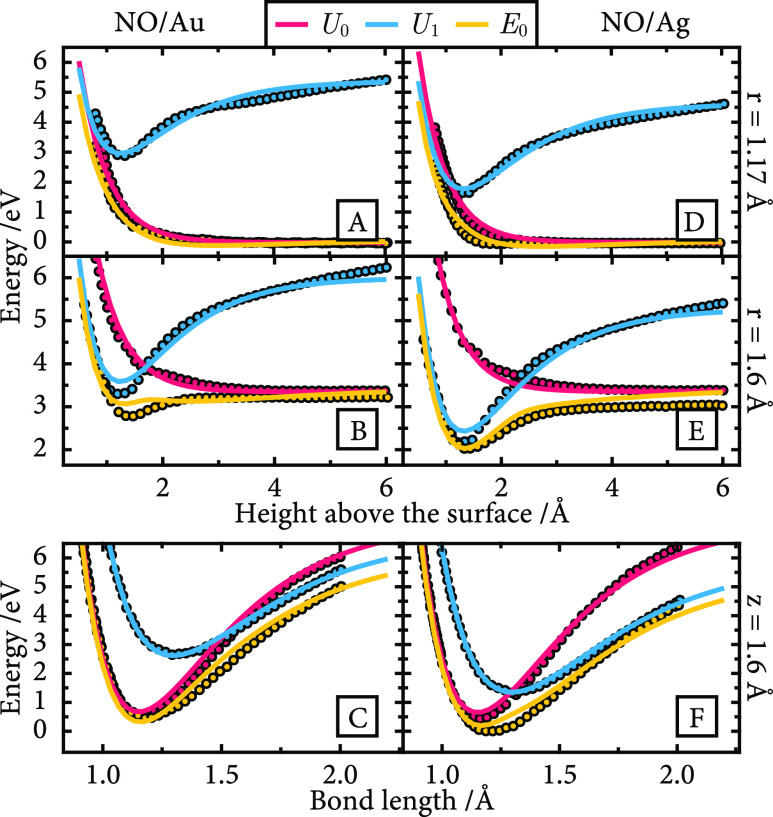
One-dimensional slices of the two diabatic potential energy surfaces *U*_0_ and *U*_1_, and the adiabatic ground-state energy *E*_0_ for the two models (NO/Au in panels A–C and NO/Ag in panels D–F). The analytic models are shown with solid lines, and the dots show the density functional theory data from ref (56) used to create the models. In panels A–E, the molecule has the fixed bond lengths labeled on the right side of the figure. In panels C and F, the molecule has a fixed height above the surface of 1.6 Å.

The resulting *U*_0_ and *U*_1_ functions are shown in Figure 1. Generally, the choice of functional form appears suitable for capturing the shape of each of the diabatic curves, although not providing a perfect fit in some areas. Some significant qualitative differences exist for *U*_1_ in panels B and E, where the depths of the minima are slightly underestimated. However, our goal is to obtain only a simple, physically motivated model where a quantitative match with the density functional theory energetics across the potential energy surface is not required. An important feature captured by the models is that NO on Ag(111) has a reduced energy gap between the neutral and anionic diabatic states, *U*_0_ and *U*_1_, compared to Au(111). This is caused by the fact that Ag(111) has a lower work function than Au(111), facilitating energy transfer from the metal to the molecule. This leads to a crossing of the diabats at a reduced bond length for NO on Ag and an enhancement of nonadiabatic electron transfer.^56^

The coupling function in eq 19 describes a monotonic decay as the molecule moves away from the surface. Since there are only two parameters, their values are simply chosen in order to best recover the adiabatic density functional theory ground-state potential energy surface, also shown in Figure 1 as a yellow curve. To keep the models as simple as possible, the coupling parameters are chosen to be the same for both the NO/Ag and NO/Au models. For both systems, the qualitative agreement between the analytic models and the reference data is good. The full set of parameters for eqs 16–19 is given in Table 1.

**Table 1:** Parameters for the Two NO Models^a^

NO Morse	
*r*_0_	1.1510 Å
*a*_0_	2.7968 Å^–1^
*D*_0_	6.610 eV

coupling	
Γ	1.5 eV
*V_k_*	(Γ/2π)^1/2^
*ã*	10 Å

NO/Au	
*b*_0_	1.9535 Å^–1^
*z*_0_	–0.26876 Å
*c*_0_	6.5713 eV
*a*_1_	2.5194 Å^–1^
*r*_1_	1.2950 Å
*D*_1_	4.1528 eV
*a*_2_	1.0015 Å^–1^
*z*_1_	1.2350 Å
*D*_2_	2.4171 eV
*c*_1_	8.9587 eV

NO/Ag	
*b*_0_	2.0402 Å^–1^
*z*_0_	–0.21164 Å
*c*_0_	6.5804 eV
*a*_1_	2.4062 Å^–1^
*r*_1_	1.2963 Å
*D*_1_	4.5879 eV
*a*_2_	0.92289 Å^–1^
*z*_1_	1.3161 Å
*D*_2_	2.8481 eV
*c*_1_	8.6327 eV

a The values for the NO Morse potential and coupling function are shared by both models.

**Figure 2:**
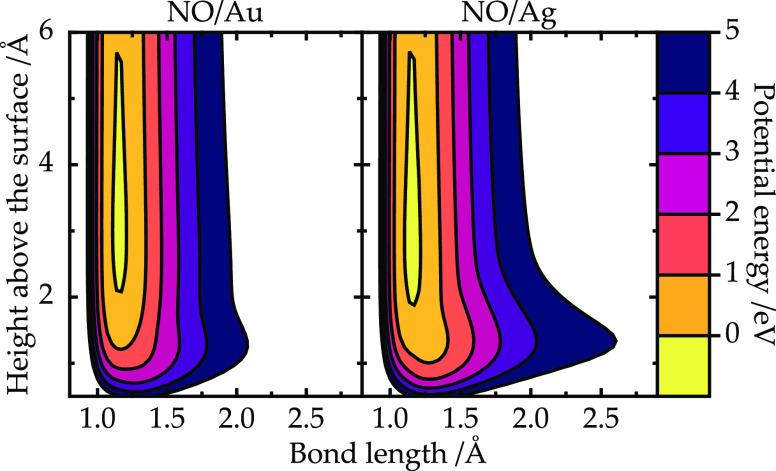
NO/Au and NO/Ag adiabatic ground-state potential energy surfaces.

The resulting ground-state potential energy surfaces are shown in Figure 2. In the entrance channel, both appear similar, but as the molecule approaches the surface, there is a softening of the bond stretching potential that is stronger for Ag than that for Au. The potential energy landscapes remain comparable at short bond lengths near the surface, where the neutral state is lower in energy, but for NO/Ag, the softening of the bond stretching potential is more pronounced. This is consistent with the crossing of the diabatic surfaces at a shorter bond length *r*.

### 3.2 Computational Details

The results presented in Sections 3.3−3.5 are obtained from molecular scattering simulations using the models in Section 3.1 and the methods introduced in Section 2.2. The masses associated with each degree of freedom correspond to the physical mass of the NO molecule; the reduced mass was used for the bond stretching motion and the total mass for the translation along the surface–adsorbate distance. In all cases, 2000 trajectories were used for every kinetic energy and vibrational initial state. The molecule begins at a height of 5 Å in a given vibrational state initialized using the Einstein–Brillouin–Keller semiclassical quantization method for a diatomic molecule as described in ref (68). The translational velocity is set corresponding to a given kinetic energy *E_i_*/eV ∈ [0.2, 1.0]. The electronic temperature is set to 300 K, and a time step of 0.25 fs is used. The metallic bath comprises 200 states with a bandwidth of 100 eV. The bandwidth was chosen to be sufficiently wide to ensure the models exist in the wide-band limit. After the bandwidth was selected, the number of metal states was increased until convergence was obtained. This procedure has previously been described.^20^

Initializing the electronic state requires special consideration for each method. For IESH, the initial electronic populations are sampled to be consistent with the Fermi–Dirac distribution at a given electronic temperature. The Ehrenfest simulations are initialized in a similar way, where the electronic wave function is initially consistent with sampled discrete electronic populations. Unlike IESH and Ehrenfest, BCME is propagated in a diabatic representation. Therefore, the BCME simulations are initialized such that the molecular level *U*_1_ is unpopulated. In the case of MDEF, the electronic populations are simply governed by the Fermi function.

Simulations are terminated when the molecular center of mass exceeds 5 Å or the duration of the simulation reaches 1 ps. Final vibrational states are obtained using the reverse of the initial quantization procedure. In the case in which the time limit is reached, the trajectory is excluded from any vibrational analysis. The standard error in each probability value is calculated as , where *p*_i_ is each individual probability and *N* is the total number of trajectories that scatter. All simulations were carried out using the open-source software package *NQCDynamics.jl* v0.13.3.^69^ The default integration algorithms within *NQCDynamics.jl* were used for all methods. For MDEF, this is the BAOAB algorithm,^70^ for IESH and Ehrenfest, the augmented Verlet algorithm as described previously,^20^ and for BCME, the adaptive fifth-order Adams–Bashforth–Moulton method (VCABM5).^71,72^ The adaptive method used the same 0.25 fs as the initial time step, with absolute and relative error tolerances set equal to 1 × 10^–10^ in atomic units.

### 3.3 Decoherence in IESH

Trajectory surface hopping simulations suffer from the issue of overcoherence, where the coherent propagation of the electronic wave function becomes inconsistent after the bifurcation of the nuclear wavepacket.^42,73,74^ To address the issue, a collection of algorithmic modifications has been proposed, collectively referred to as decoherence corrections.^75^ These involve adapting the coherent propagation of the electronic wave function to improve the internal consistency between the nuclear and electronic subsystems. Note also that coherence is not an issue restricted to surface hopping methods, affecting other methods, including Ehrenfest dynamics. Recently, a branching correction has been proposed that can be used to improve both surface hopping and mean-field methods.^76−78^ For IESH, the importance of decoherence has previously been assessed by comparing rates and diabatic populations from decoherence-corrected IESH and Marcus theory.^19^ By adapting the augmented FSSH (AFSSH) decoherence correction^79,80^ for IESH, it was shown that the treatment of decoherence improves the simulation results of IESH by more accurately preserving detailed balance.^19^

**Figure 3:**
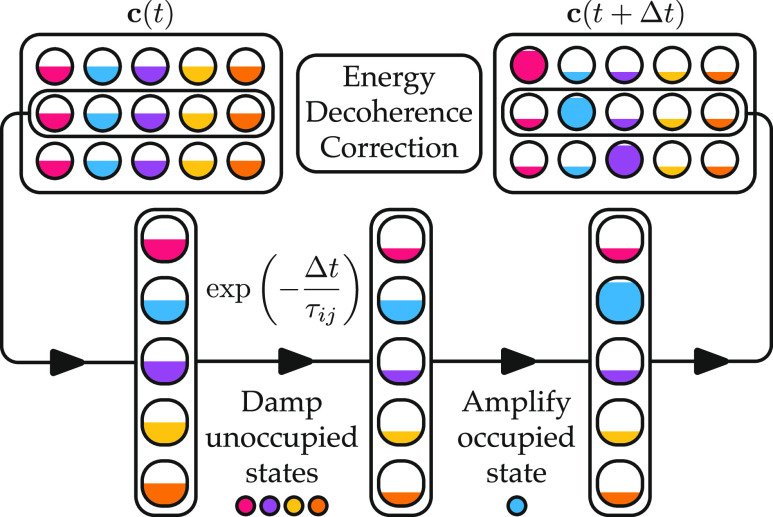
Graphical depiction of the EDC method for incorporating decoherence into the IESH algorithm using eqs 20–22. The colored objects represent the wave function coefficient for each electron in each basis state; in the diagram, there are three electrons with five basis states; each state has a different color. The fraction of the object that is colored represents the magnitude of the coefficient.

In this section, the simple energy decoherence correction (EDC) method^81,82^ is adapted for IESH and its effect on the vibrational state-to-state scattering probabilities for the NO models introduced in Section 3.1 is explored. The EDC method defines a decoherence time between electronic states *i* and *j*
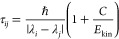
20where *E*_kin_ is the kinetic energy and *C* is a parameter set to 0.1*E*_h_.^83^ At every step, τ*_ij_* is used to damp the coefficients of the unoccupied states *c_i_* with
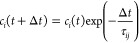
21preserving the norm of the wave function by increasing the coefficient of the occupied state *c_j_* as
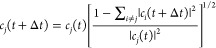
22This procedure can be extended for IESH by simply repeating the coefficient scaling for each electron in turn, such that eqs 21 and 22 are applied to the individual wave functions, using the occupations of each electron. The full version of the EDC adapted for IESH is depicted in Figure 3. The diagram shows how a single electron wave function is selected from **c**(*t*) and how the two operations that make up the EDC method are applied in turn to give the decoherence-corrected wave function. In Figure 3, eqs 20 and 21 are applied, reducing the magnitude of coefficients for the unoccupied states, and then the occupied state is amplified using eq 22. This procedure is repeated for each of the single electron wave functions.

**Figure 4:**
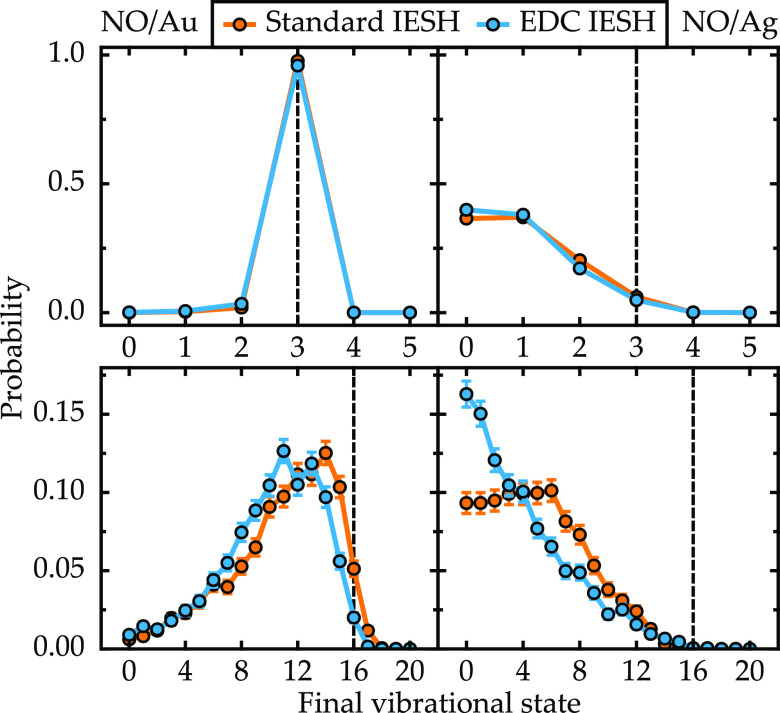
Final vibrational state probability distributions for both NO models (Au, left column and Ag, right column) using independent electron surface hopping with and without a decoherence correction. Results are shown for two initial vibrational states ν_i_ ∈ (3, 16) (top and bottom panels, respectively) with an incidence energy of 0.2 eV. The vibrational initial state is indicated by the vertical dashed lines. The error bars show the standard error associated with each point.

**Figure 5:**
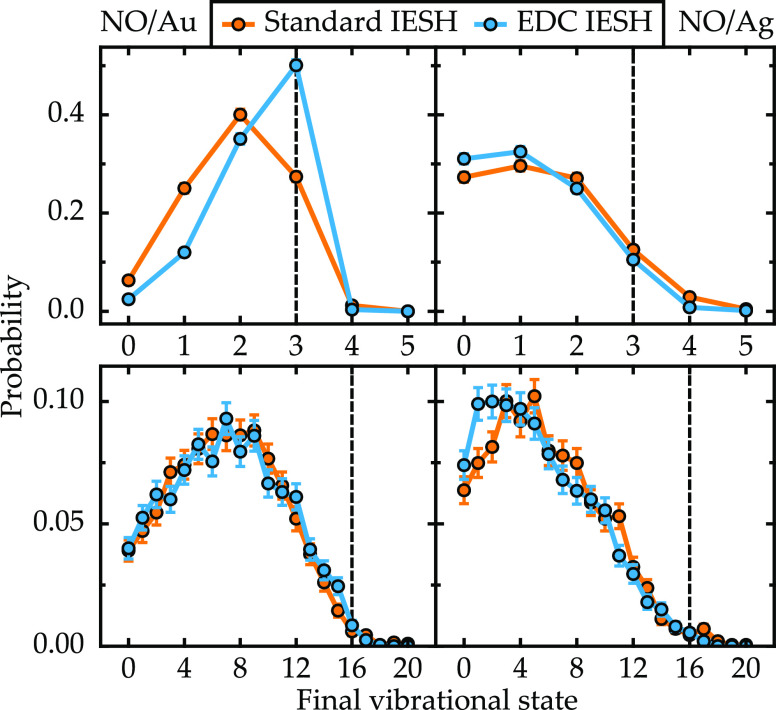
Final vibrational state probability distributions presented as in Figure 4 but here with an incidence energy of 1.0 eV.

The effect of including a decoherence correction within the IESH method is illustrated by the final state distributions presented in Figures 4 and 5. In Figure 4, the distributions obtained with low incidence energy (0.2 eV) are shown. When the vibrational energy is low (ν_i_ = 3) as in the top row, the decoherence correction has little effect on the final state distribution. However, with high vibrational energy (ν_i_ = 16), the decoherence correction changes the shape of the final state distributions. For the Au model, the peak of the distribution is shifted toward lower vibrational states. This also eliminates the small population of scattering events that have led to vibrational excitation from ν_i_ = 16 to ν_f_ = 17. In contrast, for the Ag model, vibrational de-excitation is enhanced such that the lowest-energy states are the most populated.

The corresponding results for high translational incidence energy of 1.0 eV are shown in Figure 5. In this case, even with a low vibrational energy, the decoherence correction changes the final state distribution. The most significant change is observed for Au where the vibrational de-excitation is reduced and the probability of vibrationally elastic scattering is considerably increased. In the other three cases, the effect is more subtle, only slightly adjusting the individual probabilities. In the case of high vibrational energy for Ag (lower right panel), the effect of EDC appears similar to that observed at low incidence, where the population of intermediate states (≈8) is reduced and for the lowest-energy states is increased. Overall, the decoherence correction appears to reduce nonadiabatic vibrational inelasticity for NO on Au while increasing it for NO on Ag. However, in general, it is difficult to predict how the effect of the decoherence correction is influenced by the magnitude and partitioning of the initial energy and the specific parameters of the model Hamiltonians.

In comparison with the AFSSH-modified IESH,^19^ the present EDC method has the advantage that it is simple to implement and has a negligible computational cost. To incorporate an efficient implementation of AFSSH decoherence within IESH, additional approximations to the standard method are necessary; however, for EDC, it is possible to directly use the standard algorithm without modification. In the future, it would be interesting to compare the performances of the different decoherence corrections for IESH in terms of both accuracy and computational efficiency.

The results in Figures 4 and 5 suggest that for these models the effect of decoherence is relatively subtle but can lead to quantitative deviations in the results, particularly when the energy of the projectile is high. As such, to obtain a fair comparison with the other methods, all IESH results in the subsequent sections will include the EDC modification.

### 3.4 Comparison of Mixed Quantum-Classical Methods

In this section, all methods introduced in Section 2 are applied to the models introduced in Section 3.1. The goal of these simulations is to identify how each method performs in the prediction of vibrationally inelastic scattering. However, in lieu of an exact quantum reference, it is difficult to know which method is performing best. For similar systems where the wide-band limit approximation is applied, it has been shown that BCME is able to closely reproduce the exact HQME result where quantum nuclear effects do not play a role.^84^ With this in mind, although not a perfect reference, we consider BCME as a meaningful reference to comparatively assess the performance of the other methods.

When discussing the expected performance of approximate methods for coupled molecule–metal systems, it is possible to use simple attributes of the model to estimate whether nonadiabatic effects will be significant and which methods will be most reliable. For example, the relevant quantities are often the thermal energy *k*_B_*T*, the molecule–metal coupling strength Γ, and for a harmonic system, ℏω, which provides a measure for the time scale of nuclear motion in a potential well. Comparing these quantities allows for the model to be classified and conclusions to be drawn regarding the effectiveness of each method.^26^ However, the use of the thermal energy *k*_B_*T* and nuclear frequency ℏω requires that the system be at thermal equilibrium, which is not the case during scattering simulations. Furthermore, when Γ depends on the position of the adsorbate, a straightforward comparison is no longer possible.

**Figure 6:**
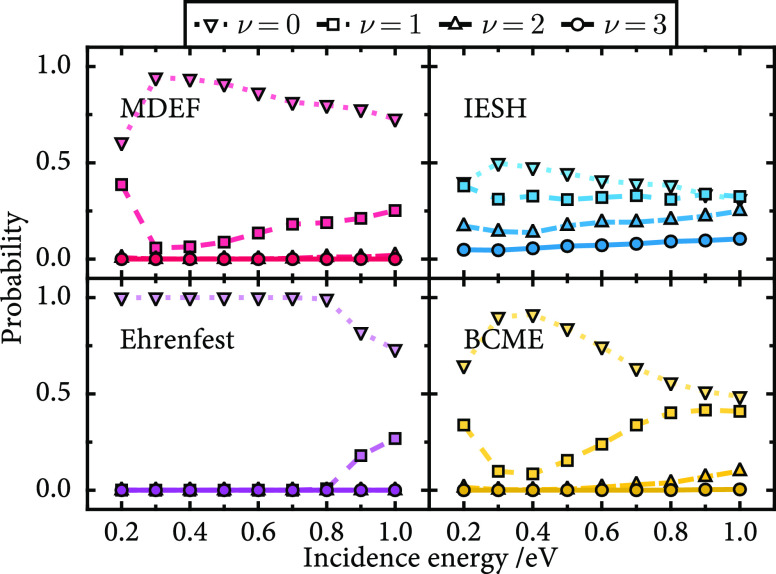
Final vibrational state probabilities as a function of incidence energy for the NO/Ag model with ν_i_ = 3. Results are shown for molecular dynamics with electronic friction, independent electron surface hopping, Ehrenfest, and broadened classical master equation. The final vibrational state probabilities are shown with markers as indicated by the legend; the corresponding lines join the markers to better illustrate the trends. The colors are used to identify each of the methods. Statistical error bars are not shown, as the error is too small to be visible.

**Figure 7:**
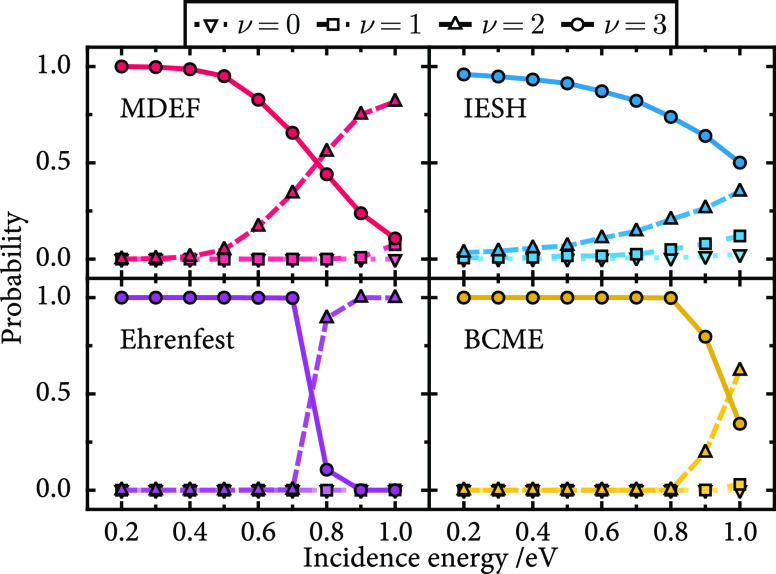
Final vibrational state probabilities as a function of incidence energy for the NO/Au model with ν_i_ = 3. Results are presented in Figure 6.

To understand how each of the methods performs, simulations have been carried out for high and low initial vibrational states with ν_i_ ∈ (3, 16) as a function of translational incidence energy. The final state distributions for ν_i_ = 3 are shown in Figures 6 and 7. We do not show the results of the adiabatic simulations as they are entirely vibrationally elastic for both models. As such, in the case of the low-dimensional models discussed here, nonadiabatic coupling is solely responsible for all vibrational de-excitation in the following results. Note that in realistic high-dimensional gas-surface dynamics, vibrational inelasticity can also occur simply due to the anharmonicity of the potential energy surface and the coupling with the substrate phonons. In most cases, all trajectories scatter successfully within the 1 ps simulation time limit; however, for some parameter combinations, a small fraction remains trapped on the surface. The proportion of these trapped trajectories is small enough (<0.01%) to be regarded as negligible.

Considering first the results for the NO/Ag model in Figure 6, it is observed that the dominant final state is ν = 0 across all incidence energies from 0.2 to 1.0 eV. This corresponds to a significant loss in vibrational energy. The results for MDEF and BCME appear most similar, with an initial increase in de-excitation from 0.2 to 0.3 eV followed by a gradual decrease as the incidence energy continues to increase. Of the four methods, IESH is the most distinct, returning a broad distribution that is largely independent of incidence energy in all four channels. Most notably, IESH is the only method that gives a nonzero probability for the vibrational elastic channel with a probability of ≈0.1. On the other hand, the Ehrenfest dynamics method yields a vibrationally cold final state of ν = 0 up to 0.9 eV. Compared to all other methods, Ehrenfest appears to heavily overestimate nonadiabatic energy loss in this case.

For the NO/Au model (Figure 7), at low incidence energy, the scattering is vibrationally elastic, and as the incidence energy increases, the ν = 2 channel gains probability, becoming the dominant channel at high incidence for all methods except IESH. As with the NO/Ag model, the MDEF and BCME results are most similar, but here, MDEF shows a smoother transition from ν = 3 to 2 that occurs at a lower incidence kinetic energy. Ehrenfest shows a sharp transition similar to BCME but the crossover is shifted to lower incidence energy by approximately 0.2 eV. Again, IESH is the most unique, showing a gentler transition than MDEF. It is also the only method that shows a significant probability for multiquantum energy loss with a small probability for ν = 1 for incidence energies higher than 0.7 eV. The NO on the Au(111) system has been investigated both experimentally and theoretically in the past for this choice of vibrational state (ν_i_ = 3).^85^ Although a quantitative agreement is not expected due to the low dimensionality and approximate nature of the current model, we find that the kinetic energy trends in Figure 7 are consistent with the experimental result. Compared to the experimental data in Figure 3 of ref (85), we see a similar decrease in ν_f_ = 3 and a corresponding increase in ν_f_ = 1, 2 probabilities as a function of incidence energy. The most notable shortcoming of the present results is the overestimation of the vibrationally elastic channel at lower incidence kinetic energies. Likely, the low dimensionality of the model that precludes dynamical steering, mode coupling, and phonon–phonon dissipation is responsible for this.

**Figure 8:**
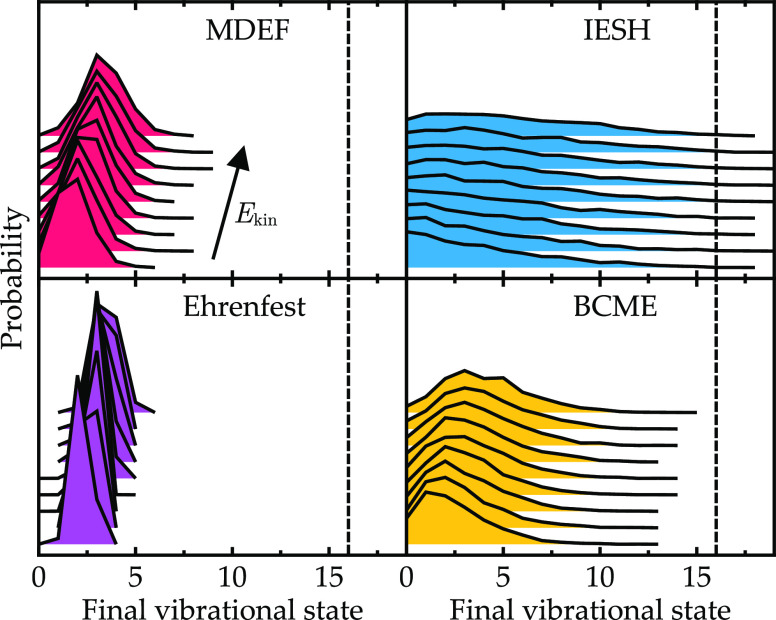
Final vibrational state distributions as a function of incidence energy, as predicted by each method for the NO/Ag model. The dashed vertical line shows the initial vibrational state (ν_i_ = 16). Distributions of increasing incidence energy *E*_kin_ are stacked on top of each other in the direction of the arrow. The incidence values range from 0.2 to 1.0 eV in increments of 0.1 eV.

**Figure 9:**
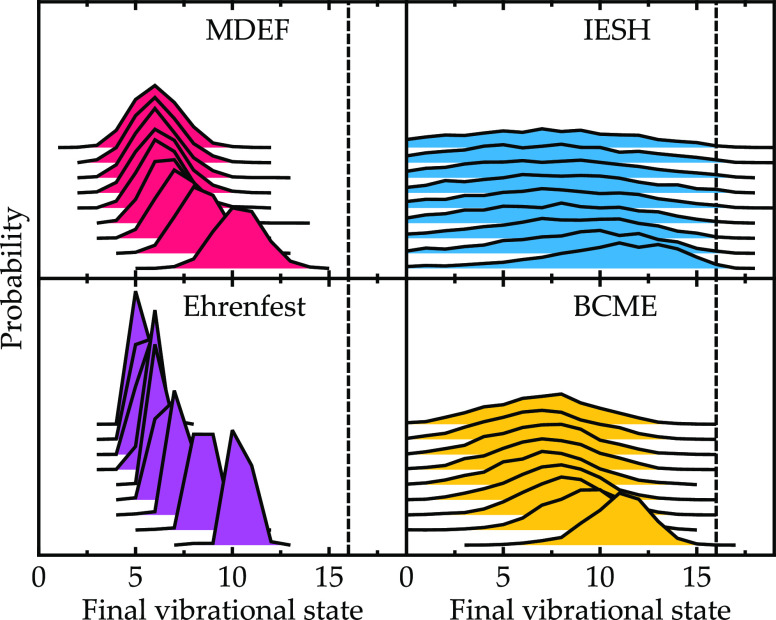
Data presented as in Figure 8 but here are for the NO/Au model.

In contrast to the low vibrational energy results, molecules prepared with a high vibrational initial state ν_i_ = 16 yield distributions that are much broader, with final states ranging from 0 up to 20. To illustrate how the final vibrational state distribution changes as a function of energy, the data are presented as a set of probability distributions in Figures 8 and 9. From these distributions, it is possible to see how the center and shape of the distributions change as a function of translational incidence energy.

For high vibrational energy ν_i_ = 16 and the NO/Ag model (Figure 8), the results for each method vary significantly, particularly in terms of the widths of the final state distributions. As the incidence energy increases, all methods follow the same trend, where the distribution shifts to higher vibrational states, with the shape of the distributions remaining mostly unchanged. Regarding the width of the distributions, it appears that the widths increase in the order Ehrenfest < MDEF < BCME < IESH. For IESH, we even find a very small population of trajectories that lead to vibrational excitation, which, although slightly suppressed by the decoherence correction introduced in Section 3.3, is unexpected for this system when compared to the BCME result. The IESH method also yields the highest energy loss with the highest point of the distribution positioned at a smaller vibrational final state when compared to the other methods. In contrast, Ehrenfest predicts a very narrow distribution of vibrational states regardless of the incidence energy.

The results for the NO/Au model with a high vibrational energy are shown in Figure 9. For this model, the incidence energy dependence is opposite to that found for the NO/Ag model, so with increasing kinetic energy, the average final vibrational state goes down and the molecule loses more vibrational energy. However, as observed for the NO/Ag model, the same trend in distribution widths is observed, with IESH overestimating the BCME width and MDEF and Ehrenfest underestimating it. The kinetic energy dependence observed for the two models can be explained with reference to the diabatic potential energy surfaces in Figure 1, specifically panels A–E, where the energy is shown as a function of height above the surface. As pointed out in Section 3.1, nonadiabatic effects are enhanced by the relative alignment of *U*_0_ and *U*_1_ for Ag(111) compared to that for Au(111). However, when the incidence kinetic energy is increased for the NO molecule on Au(111), the molecule travels closer to the surface, experiencing enhanced nonadiabatic interaction and corresponding vibrational relaxation. In contrast, for Ag(111), increasing the kinetic energy only reduces the amount of time that the molecule spends in the coupling region. The key to the different behavior is the alignment of the diabatic surfaces, where for Ag(111), they cross at a distance further from the surface and at lower energy.

The experimental result of highly vibrationally excited NO scattering on Ag(111) and Au(111) has been investigated previously (0.14 and 0.51 eV incidence energies for ν_i_ = 11 on Ag(111)^86^ and 0.5 and 1.0 eV for ν_i_ = 16 on Au(111)^30^). In the case of both Au(111) and Ag(111), the effect of the incidence kinetic energy on the final state distributions appears fairly small. Although not immediately apparent, this is consistent with the results obtained here. For Au(111), the most significant change in distribution is observed in the range of 0.2–0.5 eV; above this range, the distribution remains relatively unchanged (see Figure 9). It is exactly in this range where the experimental results are available, and the agreement is found. With Ag(111), the kinetic energy dependence remains constant across the entire range of incidence energies but is much less pronounced than in the case of Au(111) (see Figure 8). Without the experimental results for all incidence energies, it is difficult to conclude whether the model captures the translational energy dependence, but with the available data, the agreement appears satisfactory. When compared to the experimental results, it must be emphasized that the fixed molecular orientation and neglect of surface motion may lead to significant limitations. In particular, it has been shown that the initial molecular orientation (N atom facing down or O down) can influence the observed vibrational energy transfer.^13,87^ Any orientation or steering effects^15,88^ are clearly neglected by the present two-dimensional models.

Considering the results at high and low vibrational energies for both models, it appears that MDEF predicts results that are in the closest agreement with BCME, where the average final states are consistently similar. The most notable shortcoming of MDEF lies in underestimating the distribution widths for ν_i_ = 16. This is consistent with what was found for full-dimensional MDEF simulations of NO scattering on Au(111).^13^ IESH consistently has the opposite problem, overestimating the distribution widths but similarly capturing the trends in the final state. The Ehrenfest method always returns the narrowest distributions, which are clearly inconsistent with experimental findings for the systems. Both IESH and Ehrenfest are known to suffer from issues related to long-time equilibration.^18,89^ However, we do not expect these issues to significantly affect our results, as the interaction time during the scattering process is very short. Any conclusions drawn from these results have the caveat that the molecule–metal coupling strength is the same in each scenario, namely, 1.5 eV at an adsorption height of 0 Å, which corresponds to the position of the surface top layer. Only the vibrational and kinetic energies have been varied. It is hard to judge whether this is a coupling regime in which all methods can still be considered valid. Therefore, in Section 3.5, we explore artificial models with strongly reduced and increased coupling Γ to explore the limitations of the respective methods.

### 3.5 NO/Au Model with Extreme Coupling Values

**Figure 10:**
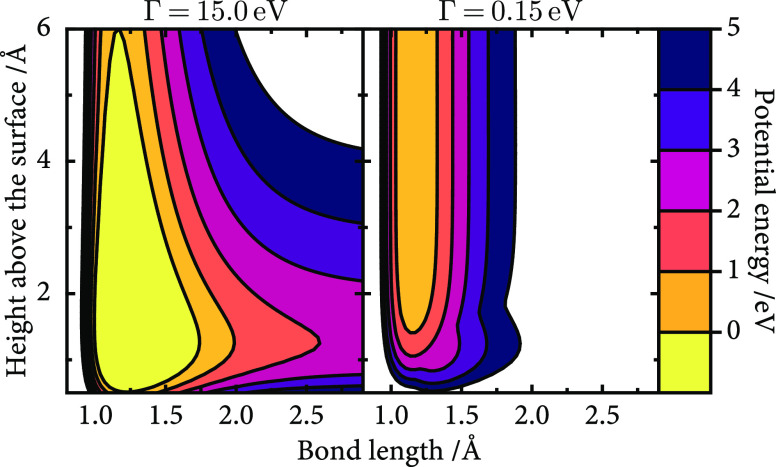
Adiabatic potential energy surface for the NO/Au model with modified coupling.

**Figure 11:**
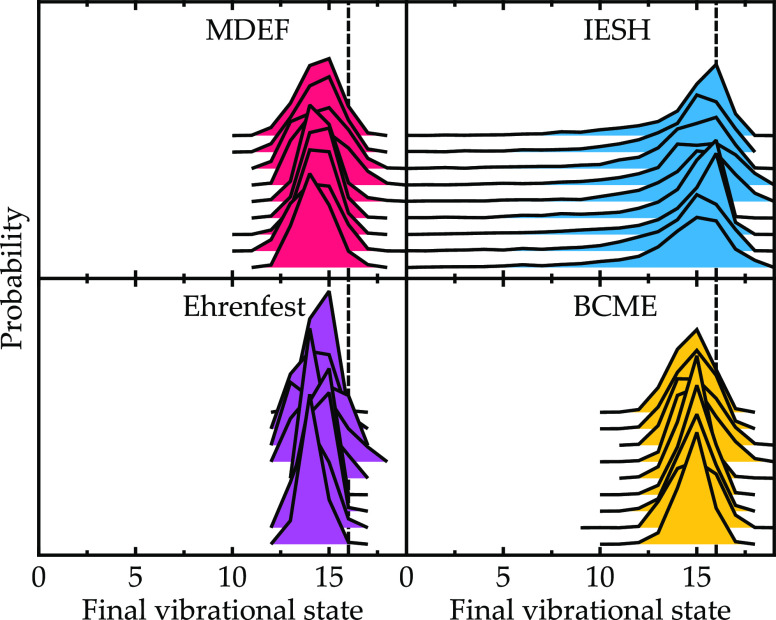
Final vibrational state distributions for the NO/Au model with increased coupling Γ = 15.0 eV presented in Figure 8.

**Figure 12:**
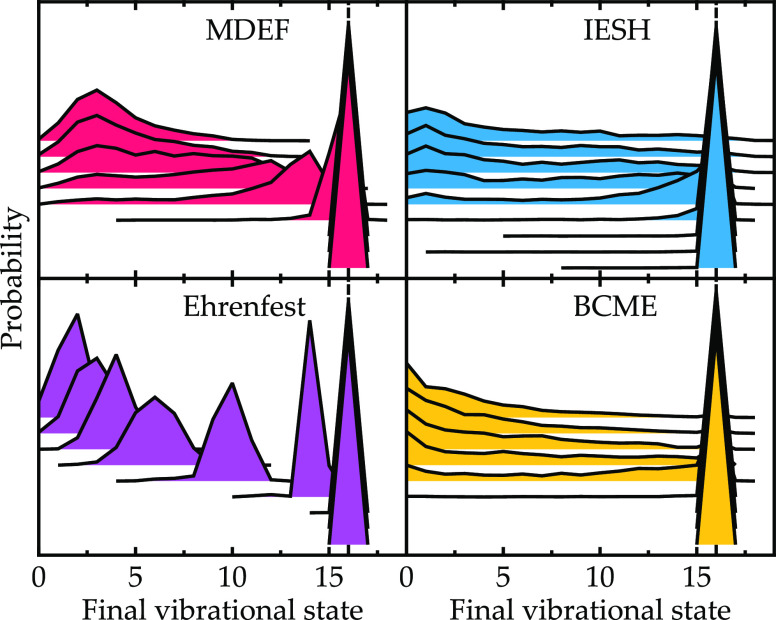
Final vibrational state distributions for the NO/Au model with reduced coupling Γ = 0.15 eV are presented as in Figure 8.

The two models introduced in Section 3.1 were chosen to have physically meaningful parameters to increase the likelihood of correspondence between the model results and the physical phenomena. However, in this section, the magnitude of the coupling Γ given in Table 1 is modified to investigate different coupling regimes. A direct scaling of Γ has the effect of altering the adiabatic ground-state potential energy surface, as we leave *U*_0_ and *U*_1_ unchanged (Figure 10). Therefore, the results of these modified models are not expected to compare to any known realistic system, and they deviate significantly from those in Section 3.4. Instead, we focus on the effect of Γ on the relative agreement between the simulation methods. To modify the coupling, Γ is scaled by a factor of 10 in both directions, with the high Γ = 15.0 eV results shown in Figure 11 and the low Γ = 0.15 eV results shown in Figure 12. Results are shown only for the modified NO/Au model. As a reminder, a low value of Γ means that the molecular state only weakly hybridizes with the continuum of metal states, and the impurity state remains a narrow feature in the density of states. For very large values of Γ, the molecular state is broadly hybridized across the electronic density of states and all metal electronic states contain a small admixture of the molecular state *U*_1_.

When the coupling is large (Figure 11), all four methods give very similar results, with only a small amount of vibrational de-excitation for all incidence energies. For this extreme coupling value, the model has entered the adiabatic regime so that each method is expected to perform well. In fact, the same result is also recovered by adiabatic MD. In the strong coupling regime, all methods are similarly capable of describing the dynamics as the role of nonadiabatic transitions is diminished.

For the model with small coupling (Figure 12), there is a sudden change in behavior, where scattering is vibrationally elastic for low kinetic energies and only becomes inelastic for *E*_kin_ > 0.4 eV. For this model, the usual trend where MDEF most closely matches BCME has changed. Now, IESH most closely matches BCME. Both MDEF and Ehrenfest are expected to work best in the (quasi-)adiabatic regime, when Γ is large, so it is not surprising that for this reduced Γ value, they do not fully capture the nonadiabatic energy loss behavior.

Having observed the results of simulations with artificially modified coupling strength, the coupling regime of the original models becomes clearer. For the strong coupling regime, we find good agreement among all methods. For the narrow coupling, we find that MDEF and Ehrenfest perform less well. This suggests that the coupling of the models fitted to the DFT results is in an intermediate regime, where the molecule–metal coupling strength gives similar time scales for nuclear and electronic motion.

To quantify the degree of nonadiabaticity in the model, we can consider the relative time scales for nuclear and electronic motion.^26^ Although Γ depends on the molecular coordinates, ℏ/Γ can be used as a rough metric for the time scale of electronic motion. For the nuclear motion, the standard harmonic approximation can be extended for the current model by including the translational kinetic energy *E*_kin_ and the vibrational state ν_i_ to give ℏ/*E*_kin_ + 1/ων_i_ for the nuclear time scale. By comparing these quantities, we can identify the adiabatic regime, with fast electronic motion compared to the nuclear motion
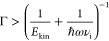
23and the nonadiabatic regime, where nuclear motion is fast compared to electronic dynamics
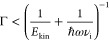
24

**Figure 13:**
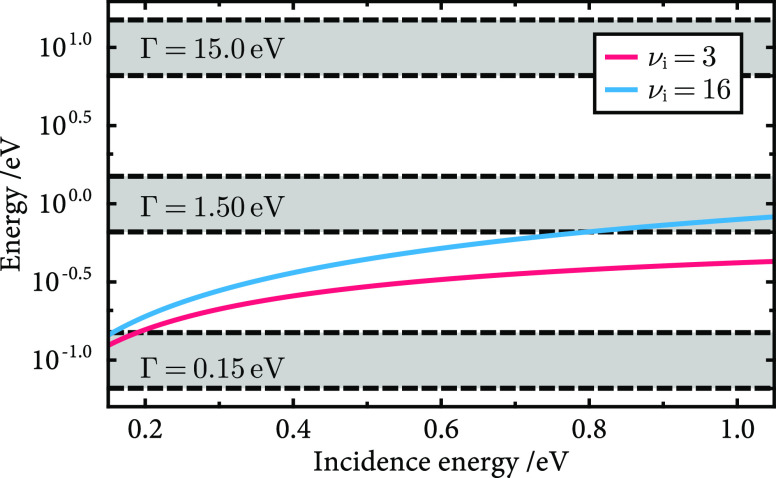
Relative magnitudes of quantities in eqs 23 and 24 for the range of incidence kinetic energies and vibrational states simulated in Sections 3.4 and 3.5. The two curves show the right side of eqs 23 and 24 as a function of the incidence kinetic energy. The shaded gray regions show the value of Γ experienced by the molecule below heights of 3.5 Å above the surface for the three coupling regimes.

Figure 13 shows the relative magnitudes of the quantities in eqs 23 and 24 for the parameters used in Sections 3.4 and 3.5. As incidence kinetic energy and initial vibrational state increase, the degree of nonadiabaticity also increases due to the increased speed of the nuclei. By comparing the relative magnitudes of the nuclear motion shown by the curves, and the range of explored Γ shown by the shaded regions, it is clear that the high Γ model exists in the adiabatic regime and the low Γ model exists in the nonadiabatic regime. For the physical models (Γ = 1.5 eV), at low incidence energy, the model appears to exist in the adiabatic regime, but as the translational energy and vibrational state increase, the relative time scales of nuclear and electronic motion become comparable. This suggests a crossover into an intermediate regime, an observation consistent with the results of the numerical simulations. Furthermore, for the NO on Au(111) system, the analysis can be used to justify previous work where it was shown that MDEF is able to describe low-energy scattering but begins to break down for high vibrational states and increased incidence kinetic energy.^13^ The success of the simple metric introduced here implies that for nonequilibrium scattering problems based on the NAH, it is possible to inform the choice of simulation method using a small selection of model parameters.

## 4 Conclusions

We have introduced two analytic models to study the vibrationally inelastic scattering of a NO molecule on two different metal surfaces, namely, Au and Ag. Using these models, we have assessed the performance of a selection of mixed quantum-classical methods, attempting to bridge the gap between simple harmonic models and full-dimensional simulations that model experiments. Within the limitations of the models, we have found that the methods predict similar trends in initial kinetic and vibrational energy dependences yet observe consistent variations in the widths of the final vibrational state distributions. Crucially, all models are able to capture important physical trends in initial kinetic and vibrational energy dependences that are consistent with the experiment and literature.

Using BCME as a reference method, we find that MDEF is reliably capable of closely matching the result for the physically motivated models (although slightly underestimating the width of the final state distributions). The IESH simulations provide relatively good agreement with BCME for high vibrational initial states but tend to provide overly broad vibrational energy loss distributions. In addition, for IESH, we have introduced a modification of the energy decoherence correction method that is able to improve the results, suggesting that decoherence effects should be considered when studying molecule–metal scattering. By modifying the magnitude of the molecule–metal coupling, we are able to establish that the model parameters extracted from previously published density functional theory data exist in an intermediate regime such that the time scales for nuclear and electronic motion are comparable. We have introduced a simple metric that uses the relative magnitudes of the incidence kinetic energy, initial vibrational state, and molecule–metal coupling to identify the regime of nonadiabaticity for models that take the form of the Newns–Anderson Hamiltonian. This metric can be used to inform decisions regarding which mixed quantum-classical methods to use in the future.

To build upon this work, the results could be verified by an exact quantum reference such as the hierarchical quantum master equation method to ensure that the BCME method is indeed a valid reference. On the topic of decoherence corrections in IESH, it will be worthwhile to investigate the relative performance of different decoherence corrections for a collection of benchmark problems. To go beyond the simple models investigated here, in the future, it may be possible to parameterize high-dimensional models more closely to ab initio data^90^ and make dynamics simulations feasible using machine learning techniques.^91^

It is hoped that this work can be used as a foundation for further tests of mixed quantum-classical methods for dynamics at surfaces. To this end, the models introduced may be used as test systems for methods that emerge in the future to comprehensively compare their performance or as starting points to explore other effects and parameter regimes. As our ability to simulate nonadiabatic dynamics of molecules on metal surfaces improves, we can better explain experimentally observed phenomena and work toward greater control of hot electron effects in chemical dynamics and catalysis at surfaces.

## Data Availability

NQCDynamics.jl is open source and available at: https://github.com/NQCD/NQCDynamics.jl. Scripts for generating the data and plotting the figures in this manuscript are available at: 10.5281/zenodo.7973913.
